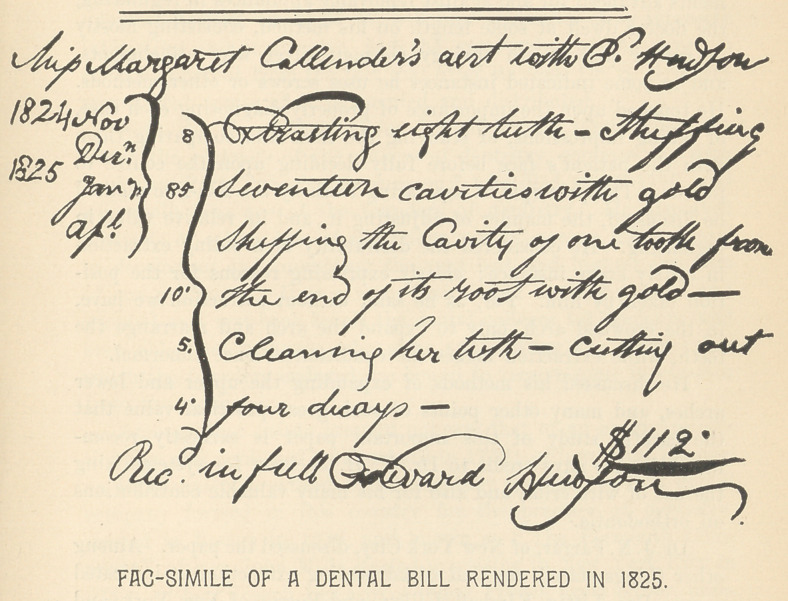# Edward Hudson

**Published:** 1902-06

**Authors:** 


### Biographical Sketch.

### EDWARD HUDSON.

EDWARD HUDSON[1] was born in 1772, in county Wexford, Ireland, his parents, it is believed, being of the religious Society of Friends. Left an orphan at an early age, he was adopted by his cousin, a dentist in the city of Dublin, who stood in the foremost rank of his profession and was a man of very considerable classical and literary attainments.

Under the kind and judicious care of this relative he was entered at Trinity College, and his studies were pursued with such ardor and delight that the result realized the fondest hopes of his adopted father.

While residing in the home of his cousin Hudson rose rapidly in qualification for his chosen profession, and practised dental

[1] Elisha Townsend, in Harris's Dictionary of Dental Science, p. 369.

surgery for a considerable time with such success that his future fame and eminence were even then confidently predicted. At college Hudson had become a prominent member of several of the debating and historical societies established about this time, and at the hospitable board of his instructor and friend he had the advantage of constant communication and intercourse with men distinguished for their literary, classical, and scientific knowledge. He thus became associated on terms of the greatest intimacy and friendship with many of the most celebrated men of his day and country,—the Emmets, Shearses, Corbetts, and Tom Moore, the poet.

The societies to which Hudson belonged at length became so open and liberal in their treatment of the political and social questions that were then agitating all Ireland that the Lord Chancellor, Clare, dissolved them and ordered the banishment of such of the members as were most obnoxious, from their pronounced views, to the government. Hudson at this time escaped the hand of authority, but immediately compromised himself still further by joining the Society of United Irishmen.

Moore,[1] in his "Memoirs," says, "Among the oldest acquaintances and friends of my father and mother were some of those who were the most deeply involved in the grand conspiracy against the government, and among the new acquaintances of the same description added this year to our list were Edward Hudson—one of the committee seized at Oliver Bond's in 1798—and the ill-fated Robert Emmet. Hudson, a remarkably fine and handsome young man, who could not have been at that time more than two- or three-and-twenty years of age, was the nephew of Hudson, a celebrated Dublin dentist. Though educated merely for the purposes of his profession, he was full of zeal and ardor for everything connected with the fine arts; drew with much taste himself, and was passionately devoted to Irish music. He had with great industry collected and transcribed all our most beautiful airs, and used to play them with much feeling on the flute. I attribute, indeed, a good deal of my own early acquaintance with our music, if not the warm interest which I have since taken in it, to the many hours I passed at this time of my life *tête-à-tête* with Edward Hudson,—now trying over the sweet melodies of our

[1] Memoirs, Journal, and Correspondence of Thomas Moore, edited by the Right Hon. Lord John Russell, M.P., p. 35.

country, now talking with indignant feeling of her sufferings and wrongs."

After his first difficulties with the government, Hudson had determined to leave Ireland and go to London in order to pursue the practice of his profession, but before he could depart, he and twenty-two other leaders of the United Irishmen were seized and transported to Fort George, in Scotland. While confined here he was visited professionally by many of the nobility and gentry of the surrounding country, and so well satisfied were they with his skill and integrity, that not only were large fees paid him very cheerfully, but great regret expressed when by his liberation from confinement his services could no longer be obtained. Being released, at the Peace of Amiens in 1802, from his long and tedious confinement of four years, Hudson abandoned his original design of settling in London and came to Philadelphia.

He began the practice of dentistry some time after his arrival in that city (probably about 1805), where he found but one gentleman who had obtained the full confidence of the public, Mr. James Gardette, a practitioner of high standing and acknowledged skill, which, together with his honesty and integrity, rendered him in every way worthy of the reputation he enjoyed.

During the early years of Dr. Hudson's residence in Philadelphia he was induced, probably by glowing accounts and representations of sudden acquisition of wealth, to engage in two distinct partnerships, neither of which seem to have been fortunate.

His first partnership was with his father-in-law, Mr. Patrick Byrne, with whom he embarked in the stationery business. This, however, was soon relinquished.

Thomas Moore was travelling in America about this time, and an extract from a letter to his mother presents another fine trait in Hudson's character,—the constancy and devotion with which he adhered in his adopted country to the republican sentiments avowed in Ireland, and which had made him a martyr and an exile from his home.

"I shall leave this place (Baltimore) for Philadelphia on to-morrow or the day after. I shall see there poor Edward Hudson, who, if I am rightly informed, has married the daughter of a very rich bookseller and is taken into partnership by the father. Surely, surely *this country* must have cured him of republicanism. . . . I have seen Edward Hudson; the rich bookseller I had heard of is Patrick Byrne, whose daughter Hudson has married; they are, I believe, doing well. I dine with them to-day. Oh, if Mrs. Merry were to know that! However, I dined with the consul-general yesterday, which makes the balance even. I feel awkward with Hudson now; he has, perhaps, had reason to confirm him in his politics, and God knows I see every reason to change mine." [1]

"It is not difficult," wrote Dr. McQuillen, "to conceive why Moore had reason to say, '*I feel awkward with Hudson now.*' Unfortunately for the poet, the Prince Regent had noticed him and the nobility of England, from the highest to the lowest, had flattered and caressed him to such an extent that they sapped his vertebral column, and he was unable to stand up before the true and sturdy associate of former days." [2]

At a subsequent period Hudson engaged in brewing, and for a time the firm seemed to be in a prosperous condition, but soon became suddenly and deeply involved, and that to an extent for which no remedy remained except the last and most trying one, the relinquishment of the whole business into the hands of the creditors. The firm paid a percentage of its debts and received a full release from all its liabilities.

It is characteristic of Hudson that he, in spite of his release, eventually paid from the income of his professional practice all that part of the original indebtedness, with interest, for which he had received the release.

After the first misfortune Hudson immediately resumed the practice of the profession which he had originally chosen, and for which he was so eminently qualified both by natural genius and education. He did not relinquish his practice on entering into the second partnership, but continued to pursue it during the whole time the connection existed, the majority of his patients and friends gladly returning to him as soon as it was known he had resumed the duties of his office. This enabled him to liquidate every claim which the unfortunate speculation had created.

From this time Dr. Hudson resumed the cheerful and bright-hearted temperament which had been habitual to him, but which, in consequence of the many and sad reverses to which he had been subjected, added to severe domestic afflictions, it was feared by his

---

[1] Memoirs, Journal and Correspondence of Moore, p. 76.

[2] Dental Cosmos, vol. iii. p. 94.

friends he had lost forever, and from this period also his professional business rapidly augmented and his circumstances became easy.

The personal appearance of Dr. Hudson was highly prepossessing. Taller than ordinary,—Moore refers to his "Herculean frame,"—his fine figure was well proportioned and graceful, and the nobility of his soul was fitly indicated by the outward grace and dignity of his bearing.

All who approached him were delighted with the blandness of his manners, his equanimity of temper, which nothing seemed ever to disturb, and those who knew him intimately respected and prized him for the exceeding goodness and sincerity which shone so brightly conspicuous in his character. By his patients he was idolized as few of his professional brethren can ever expect to be. His advice was imparted with a modest bearing which charmed, but with a quiet confidence which carried a conviction of his admirable skill to the minds of all by whom he was consulted.

Hudson's standard of excellence in dentistry was not only high but for the time somewhat novel.[1] Eleazar Parmly says of him, "We are probably more indebted to his success than to that of any other man for the importance which was attached at that period to operations which were intended to *preserve* the natural teeth in their natural state." For "by the complete success attending the practice of this great man, the public were soon convinced that teeth could be saved," instead of being extracted.

"The surgical department embracing all required operations on the living teeth . . . was as well understood and as perfectly and thoroughly practised by Hudson . . . as by any operators who ever lived, either before or since that period."[2]

In the *Dental Cosmos* for September, 1861,[3] Dr. McQuillen wrote:

"Possessing, as Hudson unquestionably did, natural endowments, extensive attainments, and an enlarged experience which eminently qualified him to contribute to the science of the profession, it is a matter of regret that he did not place something on the written page which might have advanced the interest of sci-

---

[1] Dexter's History of Dental and Oral Science, p. 14; Dental Advertiser, vol. ii. p. 3.

[2] American Journal of Dental Science, 1st series, vol. iii. p. 8.

[3] Dental Cosmos, vol. iii. p. 94.

ence and lightened the labors of less favored practitioners of his own and the present day. This remark, however, is not made in the spirit of fault-finding, for it must be admitted that one who rendered such valuable and lasting service as an operator that his work stands as an enduring monument, worthy of imitation, and his name is mentioned with a respect amounting to veneration by the community, and the profession thirty years after his body was laid in the grave, might claim exemption from the discharge of duties which, to a great extent, are regarded as binding upon those engaged in the practice of the profession at the present period. Again, isolated as he was, with few if any professional associates, he lacked that stimulus to exertion which is ever found in societies established for the cultivation of science. There can be little question, however, if the material had existed for the formation of such associations, and steps had been taken in that direction, that his ardent temperament would have carried him into such a movement *con amore,* and that his unfaltering steadiness of purpose would have kept him at his post, as an indefatigable and invaluable coadjutor, until the day of his death.

"His sphere of usefulness was extended, but it might have been boundless had he taught others how to produce the results which have placed his own name in such an enviable position in the estimation of his patients and the profession he honored."

Dr. Hudson died in January, 1833, at the age of sixty-one years, deeply lamented not only by his immediate family, but by a large circle of attached and devoted friends, among whom were numbered many of the most distinguished of the scientific and literary persons resident in Philadelphia.

In closing his biographical sketch of Dr. Hudson (from which most of the above paper is taken), Elisha Townsend says, "We are aware our words fail to do justice to the many excellencies and virtues which distinguished Dr. Hudson. To those that knew him they are not needed; to those who knew him not, what has been said may serve to give a faint idea of the character of this most excellent man and truly eminent dentist."

CHARLES MCMANUS, D.D.S.

[NOTE.—The following copy of a bill rendered by Dr. Hudson in 1825 is kindly furnished the INTERNATIONAL DENTAL JOURNAL by Dr. Emma Eames Chase, of St. Louis, the original being in her possession. This bill is interesting from the fact that Dr. Hudson charges for "*stuffing the cavity of one tooth from the end of the root with gold.*" This, as far as the writer is aware, is the first recorded account of filling root-canals.—Ed.]

## Figures and Tables

**Figure f1:**
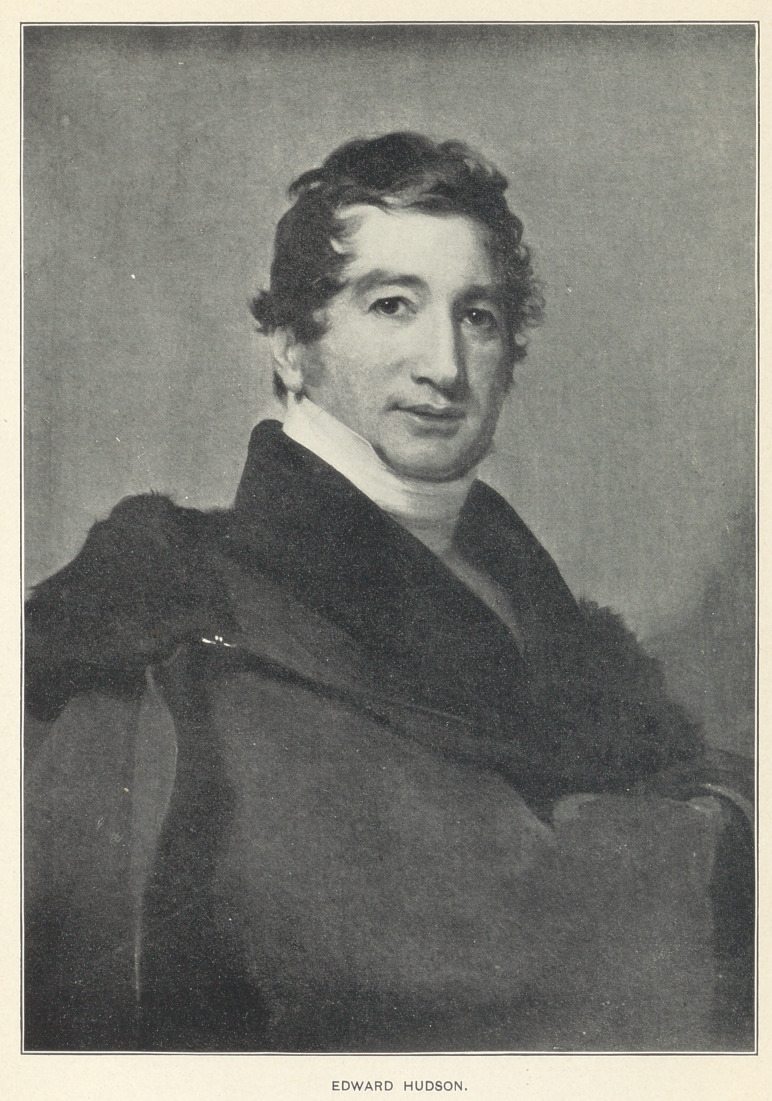


**Figure f2:**